# Assessment of novel surgical procedures using decellularised muscle and bioactive ceramic: a histological analysis

**DOI:** 10.1007/s10856-021-06585-9

**Published:** 2021-08-28

**Authors:** Randa Alfotawi, Raeesa Ahmed, Muhammad Atteya, Amer Mahmood, Abdulazize Siyal, Marium AlHindi, Ahmad El-Ghannam

**Affiliations:** 1 Oral & Maxillofacial dept, Dental Collage, King Saud University, Riyadh, Saudi Arabia; 2 College of Medicine, King Saud University, Riyadh, Saudi Arabia; 3 Department of Mechanical Engineering and Engineering Science, University of North Carolina, Chapel Hill, NC USA

## Abstract

Tissue regeneration and neovascularisation in cases of major bone loss is a challenge in maxillofacial surgery. The hypothesis of the present study is that the addition of resorbable bioactive ceramic Silica Calcium Phosphate Cement (SCPC) to Declluraized Muscle Scaffold (DSM) can expedite bone formation and maturation. Two surgical defect models were created in 18 nude transgenic mice. Group 1(*n* = 6), with a 2-mm decortication calvarial defect, was treated with a DSM/SCPC sheet over the corticated bone as an onlay then seeded with human Mesenchymal Stromal Cells hMSC in situ. In Group 2 (*n* = 6), a critical size (4 mm) calvarial defect was made and grafted with DSM/SCPC/in situ human bone marrow stromal cells (hMSCs). The control groups included Group 3 (*n* = 3) animals, with a 2-mm decortication defect treated with an onlay DSM sheet, and Group 4 (*n* = 3) animals, treated with critical size defect grafted with plain DSM. After 8 weeks, bone regeneration in various groups was evaluated using histology, immunohistochemistry and histomorphometry. New bone formation and maturation was superior in groups treated with DSM/SCPC/hMSC. The DMS/SCPC scaffold has the ability to augment and induce bone regeneration and neovascularisation in cases of major bone resorption and critical size defects.

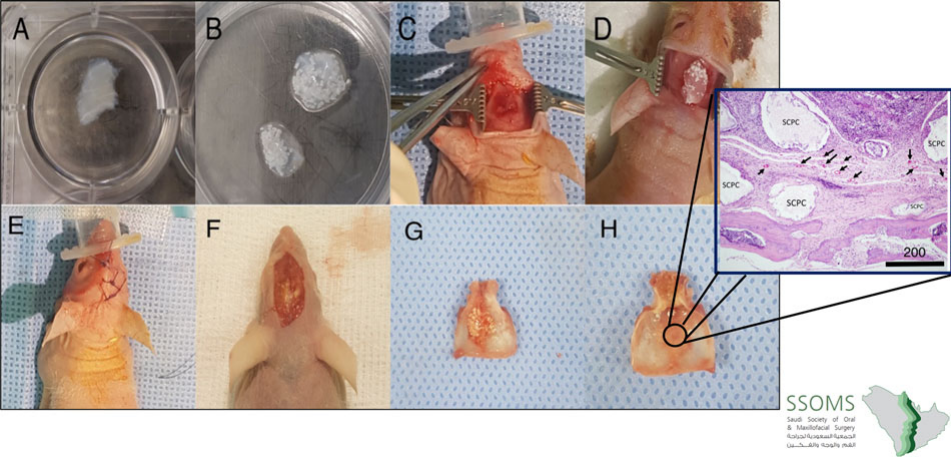

## Introduction

The use of xenografts and allografts for treatment of major bone losses and defects eliminates the donor-site morbidity and decreases the working time compared to autograft [1]. But, they are involved with the possibility of extreme immune reaction, communication of infectious diseases and slower integration with native tissue. The advancement in tissue engineering by developing skeletal muscle grafts from homologous and autologous tissues has been lately suggested as a possible option for bone replacement [2–5]. The reasoning for the utilisation of local matrix materials is the separation of site-specific extracellular matrix and gives the matrix`s proteins impressions of the previous inhabitant cells. Thus, a biomimetic scaffold can be created by decellularisation of skeletal muscle (DSM) [6, 7]. It has been demonstrated that there is a consistent cycle of exchange among cells and the extracellular framework, portrayed as unique correspondence that decides cell destiny and triggers the move from multiplication to structure arrangement [8]. Moreover, the muscle has a potential for bone forming; it has been demostrated that the incorporation of alloplastic material into skeletal muscle actuates bone development in vivo [9–11].

Lacking vascularity, nourishing background and the ability of tissue to survive have consistently been the major obstacle to the reconstruction of large bone deformities in the maxillofacial region [12, 13]. In this regard, a unique feature that makes muscular tissues an attractive matrix for bone regeneration is its vascularity. Previous studies demonstrated that endothelial cell colonisation of vessel muscular tissue scaffold enabled the conservation of the three-dimensional (3D) vascular network after implantation [6]. Thus, in attempts to overcome the problem of neovascularisation, many studies have suggested the utilisation of skeletal muscle as a supporting scaffold to influence bone regeneration and supply the necessary vascularity [4, 14–16].

Previous studies have demonstrated that exogenous sources of calcium and silicate ions strongly stimulate ossification of the newly formed bone matrix. The upregulation of osteoblast differentiation markers, tissue mineralisation and vascularisation was significantly increased in response to increased silica content in bioceramic bone graft [17–21]. New bone formation and resorption of graft material of critical size mandibular defects in a canine model were reported 4 months post grafting with silica-calcium phosphate composite (SCPC) [22, 23].

The current analysis, reports the successful use of a decellularised skeletal muscle (DSM)-SCPC as a scaffold for bone augmentation in two surgical defect models in mouse calvaria.

## Materials and methods

Transgenic nude mice (*n* = 18), weighing 24–26 g, were requested from the College of Food and Agriculture Sciences, King Saud University (KSU), then utilised for the experiment.

The ethical committee of the Institutional Research Ethics Board (IRB) of the KSU and College of Dentistry Research Centre (CDRC) reviewed and accepted the study protocol. The investigation was carried out utilising the resource and assistance of the Molecular and Cell Biology laboratory, College of Dentistry and Stem Cell Unit, College of Medicine, as a team with Prince Naif Bin Abdulaziz Health Research Centre, KSU, Riyadh, Saudi Arabia.

### Constructs preparation

The cells used were subclone derived from immortalised human bone marrow stromal cells (TERT-hMSCs) called clone cell I CLI. DSM sheets were prepared as previously reported [7]. These cells were developed by forced overexpression of the human telomerase reverse transcriptase gene in primary hBMSCs [24]. We used a TERT-hBMSC-derived subclone identified as CL1, which shows improved osteogenic, adipogenic, and chondrogenic differentiation ability. HBMSC-CL1 cells have been cultured as mentioned above.

**Fig. 1:**
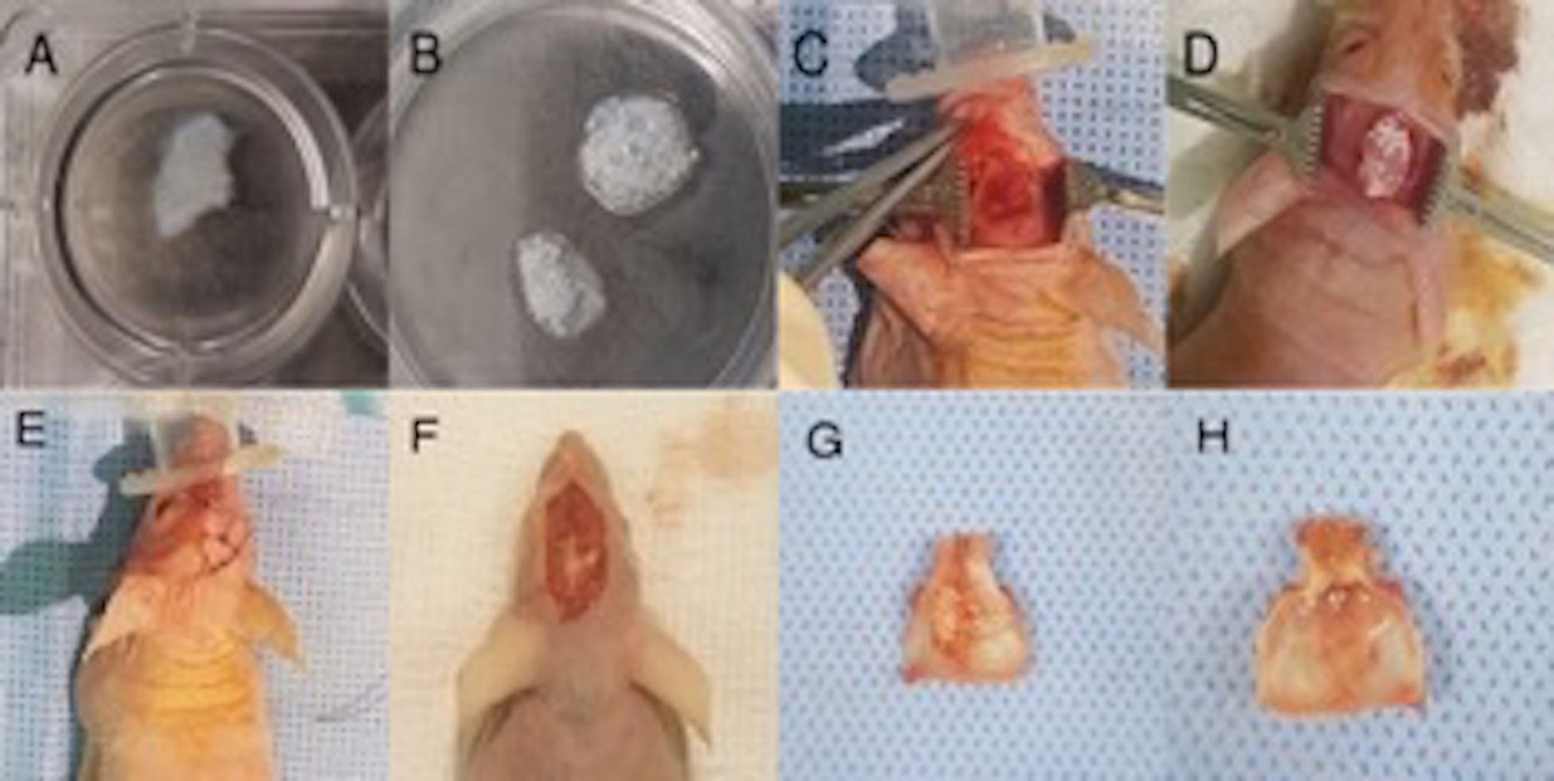
Images showing the decellularised skeletal muscle tissue adapted into the created defect of the nude mouse calvarium. **A** The muscle graft was decellularised and prepared for experiments. **B** The muscle was treated using SCPC bone cement. **C** A critical defect was created in the mouse calvarium. **D** The treated muscle was adapted into the created defect, followed by seeding of hBMSc in situ (**E**) and then the wound was sutured. **F** After 2 months, the site of the experiment is exposed. **G** The harvested calvarium bone showed remnants of SCPC and complete bone regeneration. **H** The inferior surface is inspected

Porous SCPC granules (0.7 mg) ranging from 90 to 710 μm in size (SCPC^®^, Shefabone, Charlotte, USA) were mixed with 0.5 mL of DSM culture media for 24 h before mixing with DSM. At the time of implantation, the DSM/SCPC implant was seeded with cells in situ. Characterisation of the osteogenic differentiation of the cells seeded on the decellularised muscle-bioactive SCPC hybrid was performed following the same protocol reported from our laboratory [7]. The Digital images of the DSM and the DSM/SCPC hybrid are shown in Fig. 1A, B.

### Surgery

Vaporised isoflurane anaesthesia was performed via mask inhalation at concentrations of up to 4% in the induction phase, and 0.8–1.3% during the surgery. Isoflurane was vaporised in a N_2_O/O_2_ mixture and saturated with 32–36% F_i_O_2._ The implant was prepared before creating the craniotomy defect, by mixing 0.7 mg SCPC into 0.5 cm^3^ DSM and seeding with 2 × 10^6^ hMSCs (*N* = 12). A midline sagittal incision of 8 mm was made over the cranium, followed by careful elevation of the periosteum. For animals in Group 1 (*n* = 6), multiple (2 mm) biocortical defects were made in the centre of the parietal bone using 18 gauge needles with continuous irrigation using sterile saline. For animals in Group 2 (*n* = 6), a full critical size defect (4 mm diameter) was made at the centre of the parietal bone. Control Group 3 animals had multiple 2 mm biocortical defect at the centre of the parietal bone grafted with plain DSM. Control Group 4 animals had a 4-mm calvarial defect grafted with plain DSM. One mouse was used as a negative control to assess non-operated bone. The procedure was done carefully, by not damaging the underlying dura mater thereby promoting good blood supply. The defects in groups 1 and 3 were then covered with the DSM/SCPC/hBMSC and DSM onlay, respectively. The defects in groups 2 and 4 were filled with DSM/SCPC/hBMSC and DSM, respectively (Fig. 1). The periosteum was sacrificed, and eventually closure of the surgical wound was done in layers with 4–0 Vicryl sutures. Prophylactic antibiotic coverage was achieved using an Oxytetracycline solution (10%, by injection at 0.2 mL/kg). Postoperatively subcutaneous administration of pain medication meloxicam, 0.2 mg/kg was done. Following full recovery, the mice were moved to a usual holding enclosure and 10 mL of saline was given subcutaneously (SC) during the post-operative care to prevent dehydration. The activities of the mice and the surgical site healing were checked consistently.

Following 8 weeks all mice were euthanized by the use of highly concentrated CO_2_ in glass chambers after the craniotomy was performed. As demonstrated by losing the righting reflex, the mice were unconsciousness as the level CO_2_ rose from 40 to 50%. Now, the progression of gas was expanded to quickly fill the chamber, minimising the time to death. The CO_2_ stream was sustained for at least 1 min following respiratory arrest. The abdominal fat tissues and adjacent soft tissues were collected and fixed in 10% formalin for histological analysis.

The cranial defect areas, along with the adjacent bones and soft tissues, were harvested. The samples obtained were set at 10% formalin for histological analysis.

### Histological analysis

The samples were moved to plastic holders with 10% buffered formalin. The regenerated tissue and the adjacent native bone were divided into upper, middle, and lower segments. The samples were taken out from the 10% formalin, stacked into cassettes of appropriate size, and put in a rotor basket buffered in 10% formic acid for decalcification. Fluoroscopy was used to verify the end point of the decalcification procedure in order to prevent undue tissue injury. Sections of 5-µm were prepared from the decalcified tissues, which was immersed in paraffin wax to form blocks. Haematoxylin and eosin (H & E) and Masson trichrome stains were used to stain the sections. Slides were then studied under a light microscope (Olymp us, Japan) (Zeiss, Germany) supplemented with an Axio-Vision camera.

### Immunohistochemistry and histomorphometry

Immunostaining of the paraffin portions of the decalcified tissue to detect osteopontin was conducted utilising the streptavidin-biotinylated horseradish peroxidase (S-ABC) process (Novalink Max Polymer detection system, Novocastra, product NO. RE7280-K). The methods followed were: 3% H_2_O_2_ in distilled water for a duration of 5 min was used to inhibit the action of endogenous peroxidase, afterward they were washed twice in Tris-buffered saline (TRS) (Sigma, T 5030-100 TAB, pH 7.6) for 5 min each. Incubation with a protein block for a duration of 5 min (Novocastra) was used to block non-specific bonding to the antibody. Rabbit anti-osteopontin (dilution of 1:200) (Cat# Sigma-Aldrich 07264) was used to incubate the sections. Slides were incubated with primary antibodies at room temperature for 1 h. They were washed three times in TRS for 3 min per time, followed by incubation with biotinylated anti-rabbit IgG (Novocastra) for 30 min. Next, they were washed in TBS three times for 3 min each, later incubating them with Novolink polymer (Novocastra) for 30 min. Then, the washing in TBS three times for 3 min each was repeated. Peroxidase has been observed with a working solution of diaminobenzidine (DAB) substrate (Novocastra) for 10 min. Finally, distilled water was used for 10 min to wash them. The sections were then counterstained with Mayer’s haematoxylin and mounted in dysterene, plasticiser, and xylene (DPX). In the case of negative control sections, same method was applied except with no incubation of primary antibodies.

### Image analysis

High-resolution full-slide optical scans of the anti-osteopontin immunostained parts were produced using the ScanScope scanner (Aperio Technologies, Inc.). Later the digital slide images were seen and analyzed utilising the viewing and image processing methods of Aperio’s ImageScope program (Aperio Technologies, Inc.). Each section was divided into three regions: centre of defect, interface, and newly mineralised bone (either at the centre or at the border). Random selection of five regions, each with a fixed size of 19,200 µm^2^ per zone was done. Colour deconvolution (colour separation) programme (Aperio Technologies, Inc.) was fabricated (by colour calibration) to measure the immunopositive response and to identify and evaluate only the brown colour of DAB-positive staining. The programme was then applied to the chosen region to calculate the proportion of immunopositive reactions compared to the overall area of study. The thickness (µm) of newly formed bone in the centre of the deformity was measured. The results obtained from the image analysis were sent for statistical analysis.

### Statistical analysis

The data obtained was analyzed using IBM SPSS Statistics, Version 22. The normality and homogeneity of data variances were tested by the Shapiro–Wilk test and the Levene test, respectively. For an overall comparison between the groups tested, One-way analysis of variance (ANOVA) and the Tukey highest significant difference (HSD) test were used. The post-hoc pairwise comparisons was done by the Scheffe test. When *p* value was 0.05 or less, significance was taken into consideration.

## Results

### Clinical examination

**Fig. 2:**
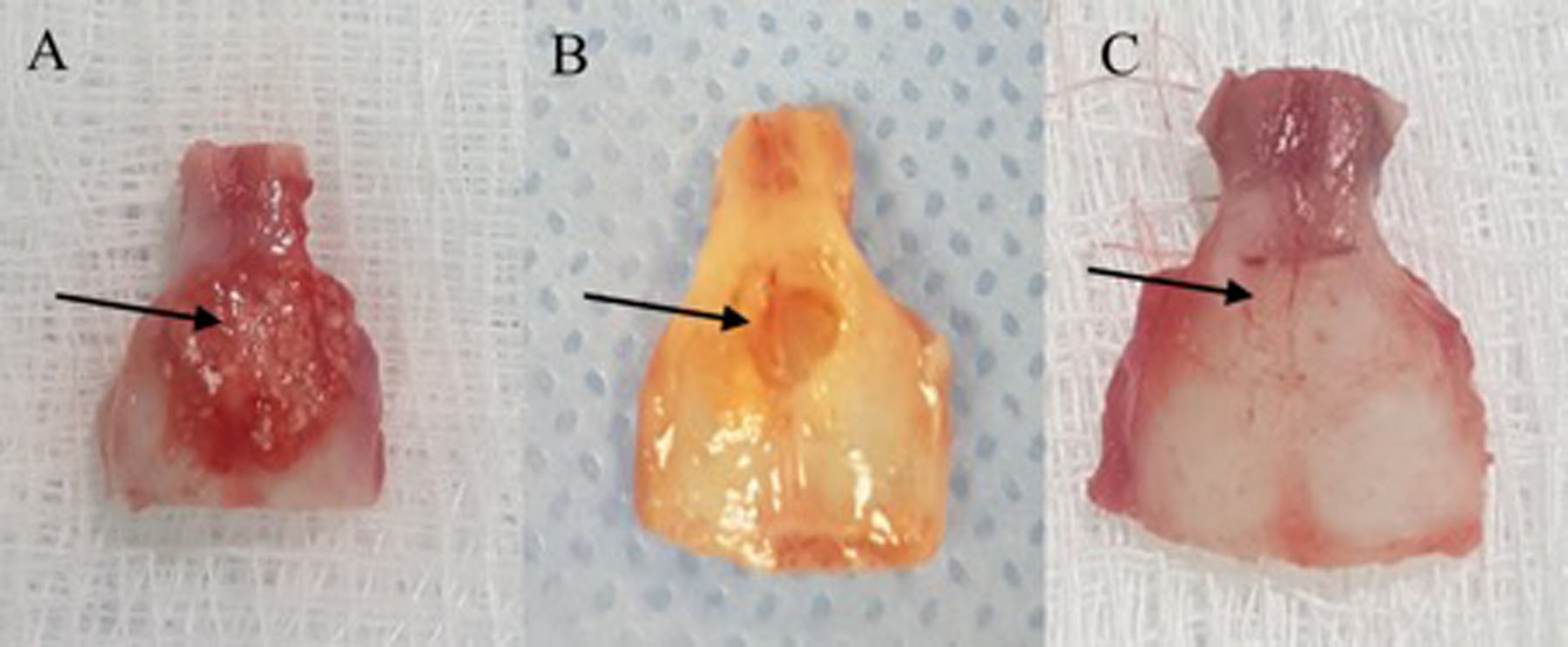
Different harvested calvarial bone from the tested groups. **A** Group 1: corticated bone grafted with SCPC/DMS/hBMSC, **B** Group 2: critical size defect grafted with SCPC/DMS/hBMSC, and **C** Group 3: corticated bone grafted with DMS alone

The cranial bone samples from Group 1 showed graft material remnants that is SCPC particles surrounded by membrane-like structures (Figs.1F and 2A). The residual tissues are representative of a defect on the outer surface of the defective region. However, total tissue regeneration was seen in the inner surface of the defective area (Fig. 1H). Whereas, in Group 2, the critical size defect showed thin translucent tissue that looked like bone, and no remnant of SCPC was seen in any of the samples (Fig. 2B.) In contrast, the positive control samples (plain decellularised muscle) did not show remnants of decellularised muscle but a complete bone covering the defect was observed (Fig. 2C).

### Bone healing in corticoid cranial defect

**Fig. 3:**
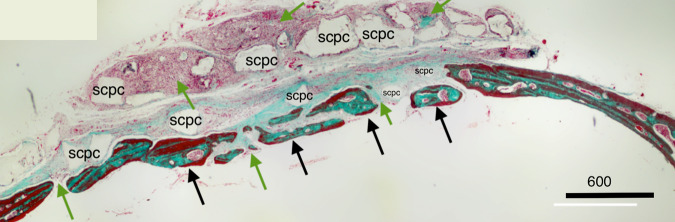
Photomicrographs showing decalcified histological sections of Group 1 (DSM/SCPC/hBMSC) stained with Masson trichrome. The sections show the whole cranial defect area with formation of new bone inside the defects (green arrows). The black arrows point at the host bone and remnant of the SCPC granules

**Fig. 4:**
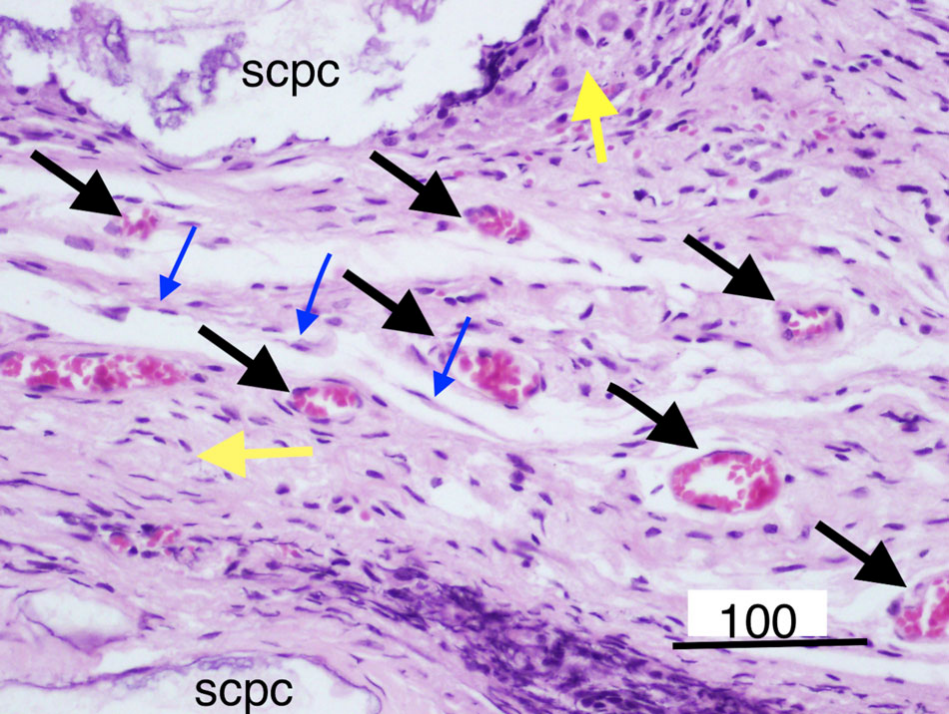
Photomicrographs showing decalcified histological sections of Group 1 (DSM/SCPC/hBMSC) stained with H&E. Areas of bone growth within the corticoid defect as well as the onlay graft are evident. New woven bone bridges the defect and grows upward. Remnant SCPC granules can be seen with high cellular activity around them and direct bone deposition on the material surface. Numerous blood vessels (black arrows) are recognised close to the SCPC particles. Islands of mature bone can be seen surrounded by high cellular activity and immature bone and loose connective tissue as periosteium-like structure (blue arrow), Scale bar = 100 μm

**Fig. 5:**
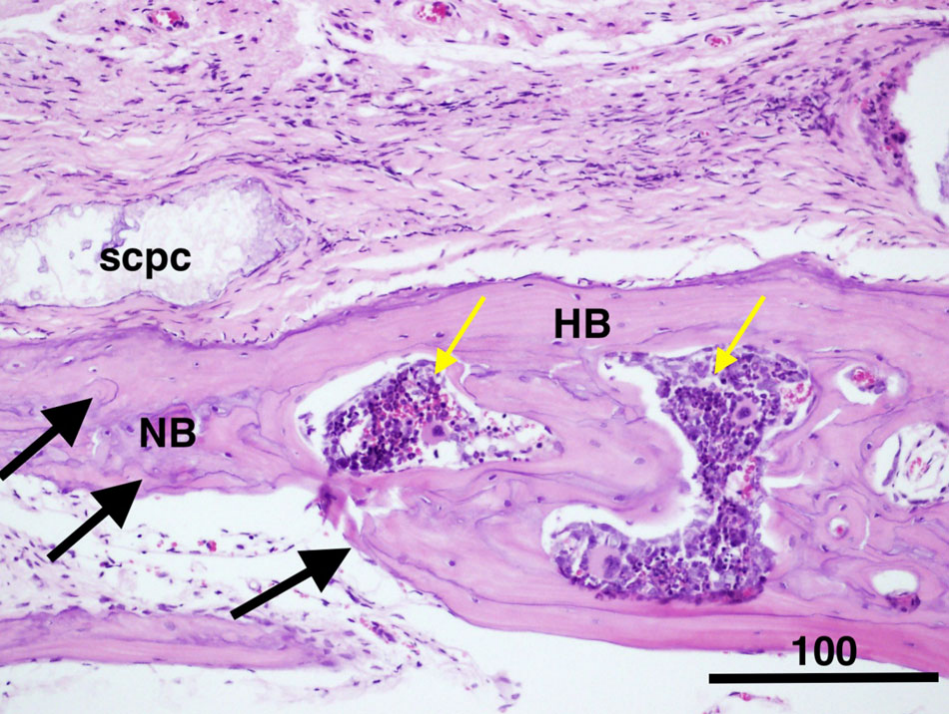
Photomicrographs showing decalcified histological sections of Group 1 (DSM/SCPC/hBMSC) stained with H&E. The integration between the newly formed bone and the host bone. The presence of the reversal lines in the newly formed bone denoted with a black arrow; large marrow spaces are denoted with a yellow arrow, scale bar = 100 μm

Masson trichrome staining (Fig. 3) of decorticoid cranial defects treated with the DSM/SCPC/hBMSC onlay sheet showed formation of new bone inside the defects. The newly formed bone has faint red staining indicating less maturation than the deep red stained host mature bone. Formation of new immature bone was also noted throughout the entire thickness of the onlay sheet. A periosteum-like membrane from loose collagenous tissue separated the remnants of DSM from each other (Fig. 4). Remnant of the SCPC granules could be seen (Fig. 4) surrounded by areas of high osteoblastic cellular activities, indicating the progress of bone formation. Numerous blood vessels (Fig. 4) were also seen near the SCPC particles and area of immature woven bone matrix. The newly regenerated bone is well incorporated into the host bone (Fig. 5).

**Fig. 6:**
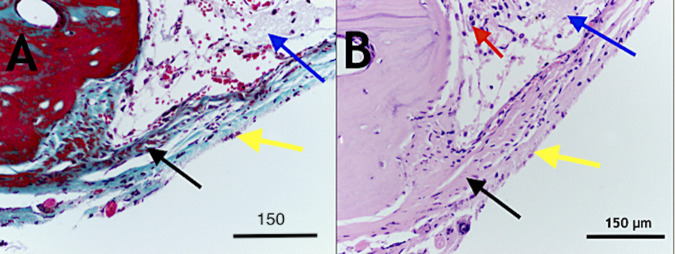
Photomicrograph showing (Group 1) decalcified histological sections stained with Masson trichrome (**A**) and haematoxylin and eosin (H & E) (**B**). The area of the interface between the remnants of DSM, which became highly cellular with the appearance of osteoblasts and pre-osteoblastic cells (red arrow) in the collagenous matrix with immature bone deposits (black arrow). The DSM remnant is denoted with blue arrows; the periosteum-like structure is denoted with yellow arrows, scale bar = 150 µm

The thickness of the regenerated bone along with the adjacent host bone increased and showed displaced cortices associated with the increased trabecular bone and formation of numerous marrow spaces (Fig. 5). The presence of the reversal lines in the newly formed bone is indicating that remodelling is taking place rapidly. Next to the periosteum, a separate pattern of lamellar growth was found (Fig. 5). The interface with remnants of the graft with bone demonstrated a woven bone, a membrane like periosteum, an exceptionally cellular region, and a collagen matrix with rich pre-osteoblastic and osteoblast-like cells (Fig. 6).

**Fig. 7:**
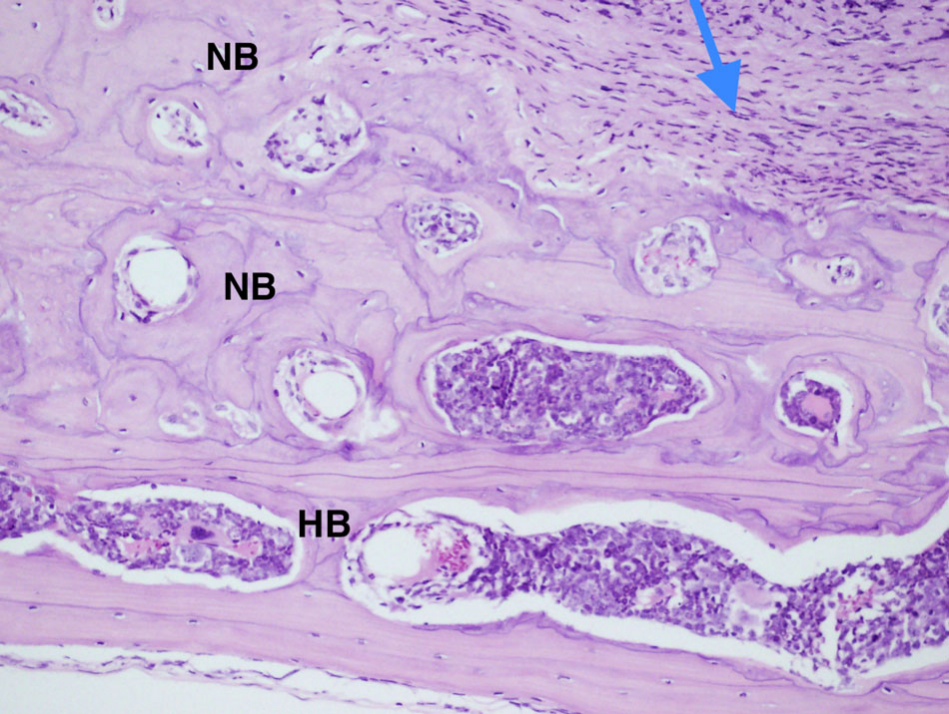
Photomicrograph showing the thickness of the host cranial bone (HB) next to the grafted tissue (blue arrow). The new bone (NB) regeneration reached more than double the thickness of non-operated bone, scale bar = 100 μm

**Fig. 8:**
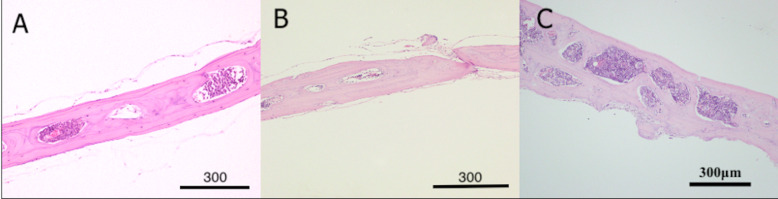
Photomicrograph showing decalcified sections of calvarial bones stained with haematoxylin and eosin (H & E). **A** Section of non-operated bone; the measured thickness = 280 µm. **B** Bone section after treatment with plain DSM; thickness = 282.80 µm. **C** Bone section after treatment with DSM/SCPC/hBMSC onlay showing double rows of fused marrow spaces and bone thickness of 400−600 µm, scale bar = 300 μm

Interestingly, thickness of the host cranial bone (HB) next to the grafted defect showed more than double the thickness of unoperated bone or bone treated with DSM alone (Fig. 7). Moreover, the thick bone (Fig. 8C) in the animals treated with DSM/SCPC/hBMSC appeared vascularised and contained numerous large bone marrow spaces full of active cells, indicating the suitability of the DSM/SCPC/hBMSC onlay sheet for bone augmentation. Conversely, in control samples (Group 3) treated with (plain DSM), a bi-cortical lamellar bone was noted in the defect with complete resorption of the DSM graft, which attained similar bone thickness to the non-operated bone (Fig. 8A, B). For Group 4 there was little change in the size of the defect it was mainly occupied with white fibrous like tissue. The bone in the border of the deformity was observed to be irregular, owing to bone regeneration or active remodelling.

The non-operated calvarian bone section of nude mice revealed a thickness of 200 μm (Fig. 8A) with several bone marrow spaces in one row along with increased remodelling rate in spite of lamella bone occupancy.

**Fig. 9:**
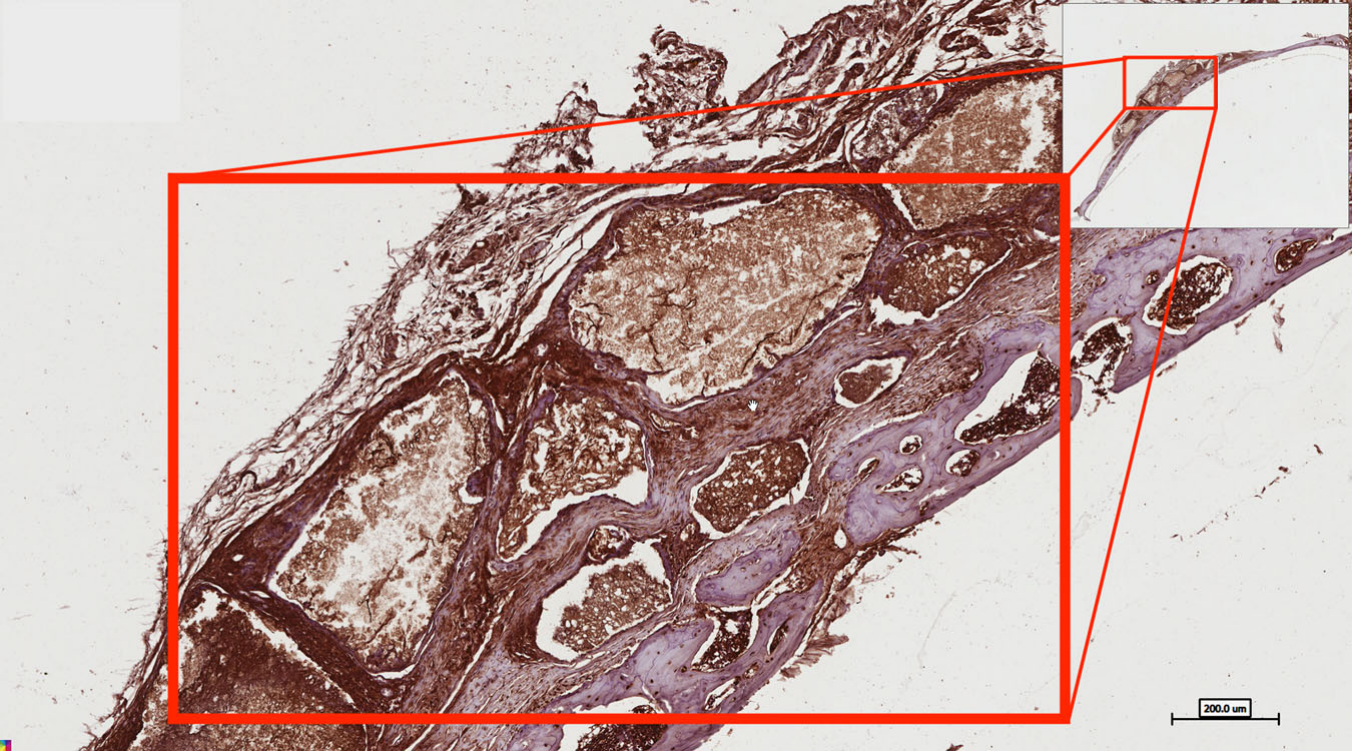
Photomicrograph showing osteopontin staining for woven bone formation through the entire thickness of the onlay DSM/SCPC/hBMSC sheet graft. The onset demonstrates direct bone formation on the surface and in between the SCPC particles. The dark staining of the cells indicates differentiated osteoblasts

**Fig. 10:**
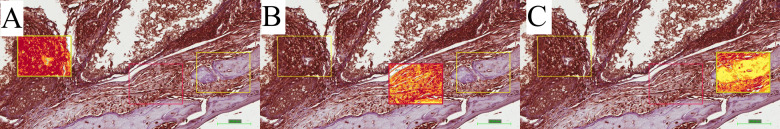
Photomicrograph showing colour deconvolution of selected areas in the cranial bone defect sections immune-stained with osteopontin. **A** Area at the centre of the defect with intensity 61.48 ± 13. **B** Area at interface of newly regenerated tissue and bone with average intensity 28.5 ± 8.52. **C** Area of newly regenerated bone with intensity 4.9 ± 4.1. Scale bar = 100 mm

**Fig. 11:**
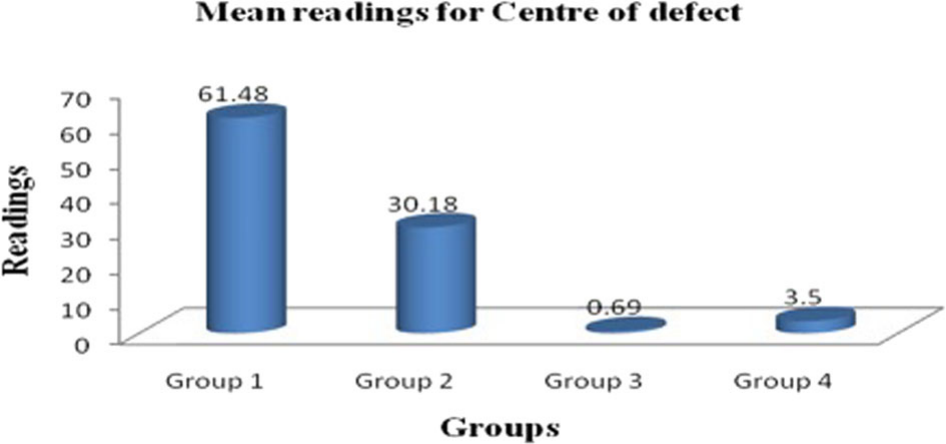
Histogram shows the main reading for osteopontin stain at the centre of the defect

Osteopontin staining confirmed the formation of bone matrix within the defect as well as through the entire thickness of the grafted onlay (Fig. 9). We used a colour deconvolution algor DMSC ithm (Aperio Technologies, Inc.) to quantify the osteopontin immunopositive reaction. The intensity of osteopontin immune staining increased in the following order: centre of the defect > interface between new and host bone > newly regenerated bone. Statistical significance was noted with this difference (*p* value < 0.001) (Table 1, Figs. 10 and 11).

**Table 1:** Multiple comparisons for centre of defect using tukey HSD

Groups	Compared with	Mean difference	*p* value
Group 1	Group 2	31.29^a^	<0.001
	Group 3	60.78^a^	<0.001
	Group 4	57.98^a^	<0.001
Group 2	Group 3	29.49^a^	0.002
	Group 4	26.68^a^	0.006
Group 3	Group 4	2.802	0.991
Group 4	Group 1	25.01^a^	<0.001
	Group 2	26.68^a^	0.006

^a^The mean difference is significant at the 0.05 level

The tissue surrounding the SCPC remnants showed highly intense brown staining, which indicates active osteoblastic activity and an area of immature bone formation (Fig. 10). Whereas, the area of newly generated mineralised bone has less stain.

### Bone regeneration in critical size defect

**Fig. 12:**
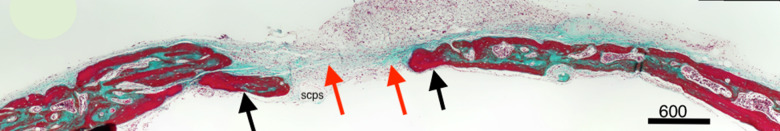
Photomicrograph of Masson trichrome staining of the critical size bone defect grafted with DSM/SCPC/hBMSC showing new bone formation and maturation. Area of bone growth from the edge of the defect is denoted with a black arrow, and area of an immature bone at the centre of the defect is denoted with a red arrow

Masson trichrome staining showed new bone formation with resorption of graft material in the critical size defect (Fig. 12). The red staining of the regenerated bone suggests bone maturation. Bone growth along with maturation began from the edges of the deformity and advanced into the centre. The centre was filled with woven bone, as is indicated by the greenish colour and characterised by high cellular activity.

**Fig. 13:**
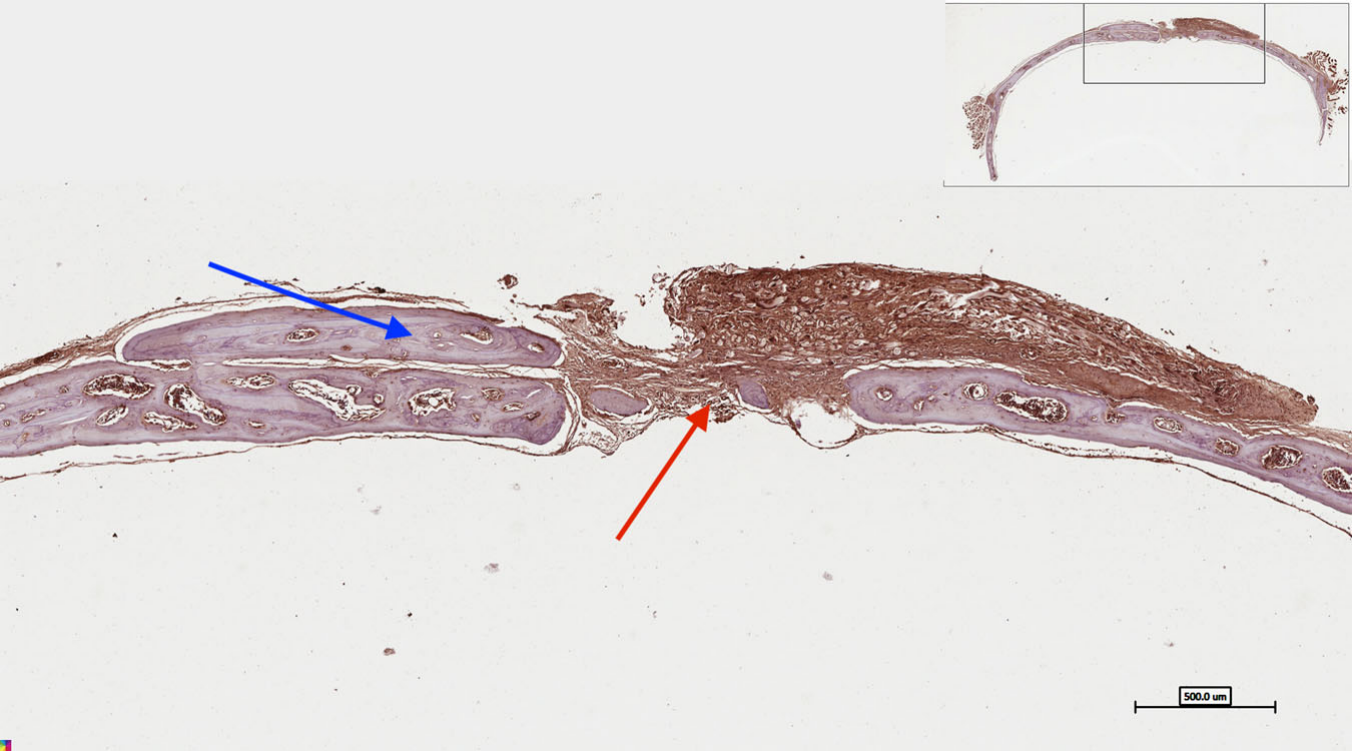
Photomicrograph of osteopontin (OPN) immunohistochemistry staining of critical size calvarial defect treated with DSM/SCPC/hBMSC

**Fig. 14:**
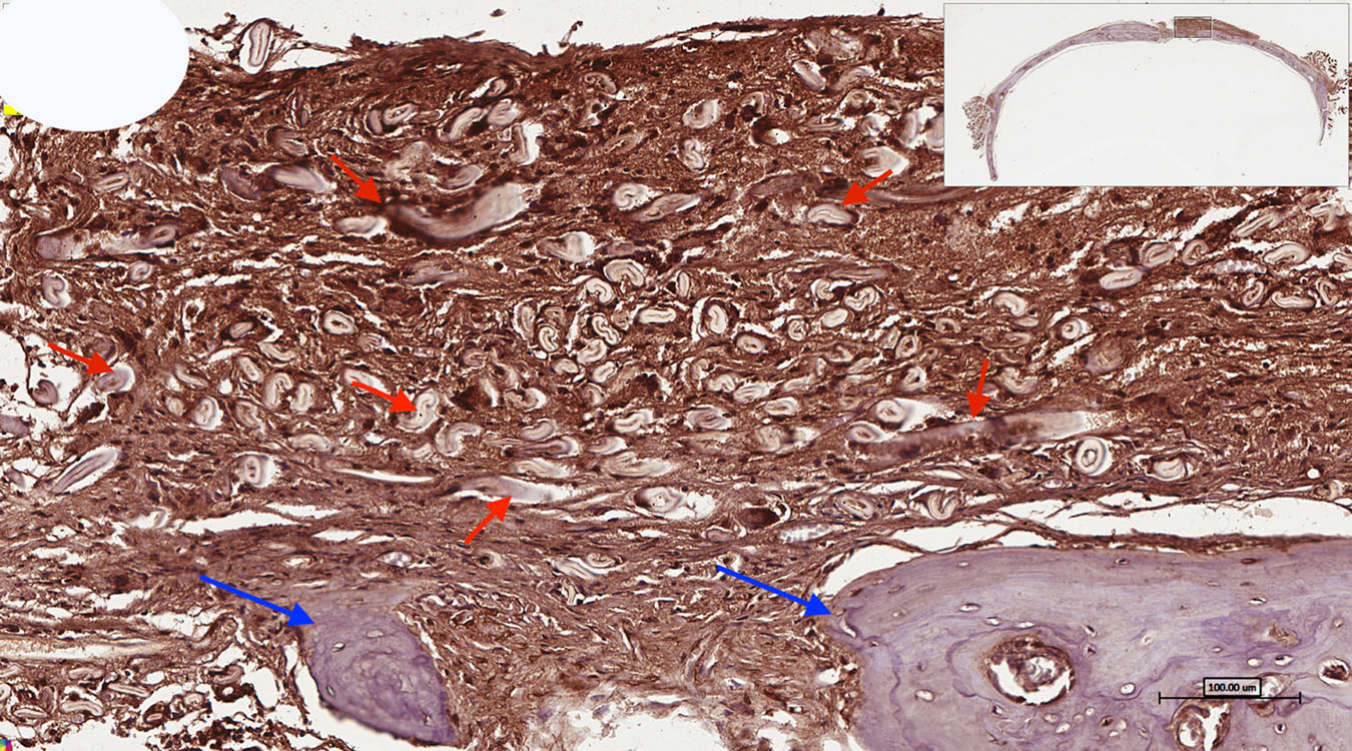
Higher magnification for the centre of the defect showing numerous bone spicules (blue arrows) surrounded with connective tissue that shows positive intake of osteopontin (OPN) immunostaining

The osteopontin (OPN) immunohistochemistry stain showed the newly formed bone stained brown with areas of woven bone and bone spicules within the centre of the deformity (Fig. 13). The regenerated bone and the adjacent host bone showed highly displaced cortices associated with the increased trabecular bone and formation of numerous marrow spaces. Higher magnification for the area of the defect is presented in Fig. 14.

**Fig. 15:**
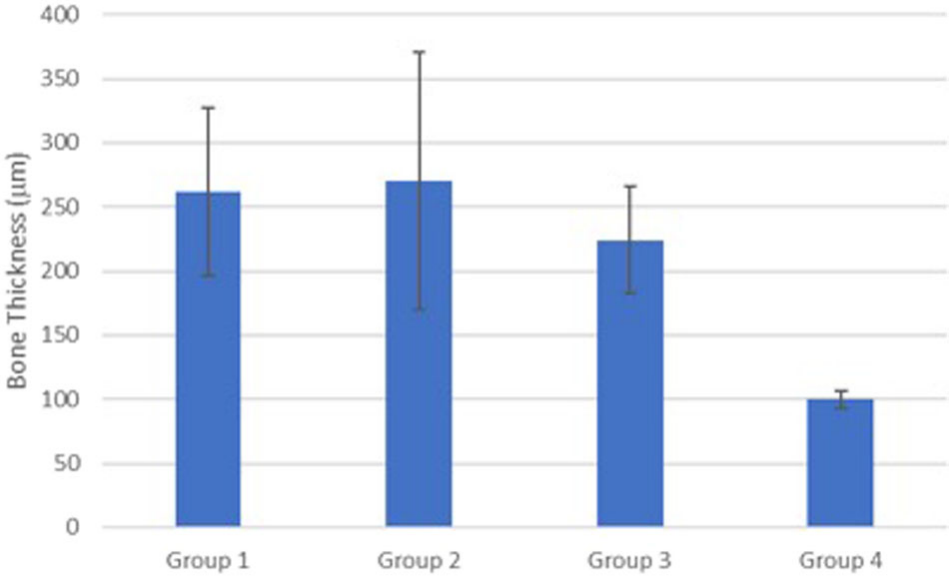
Graph comparing the mean bone thickness in all the tested groups with the thickness of native non-operated bone (100 µm). A significant difference was found at *p* value < 0.05

Comparison of bone thickness among various groups indicated significantly higher thickness for the treated grafted groups (gp1 and gp2) compared with non-operated bone (Fig. 15). When compared with the thickness of native non-operated bone (100 µm), a significant difference at *p* value < 0.05 was reported. the rationale behind this phenomenon was due to bone formation within the scaffold as well as the stimulation of cells by the dissolution products of SCPC bioceramic. One may infer the suitability of use of this construct clinically at areas that require bone augmentation.

## Discussion

The results of the study demonstrated the ability of DSM/SCPC/hBMSC to regenerate bone in two different surgical models including decorticoid and critical size calvarial defects. The DSM served as a scaffold to provide guided tissue growth, and the SCPC resorbable bioactive ceramic served as a source of Si and Ca ions necessary for enhanced vascularisation, osteoblastic differentiation and expedited bone formation [19, 20, 22, 25]. The addition of bone marrow cells in situ facilitated recellularisation of the DSM/SCPC hybrid with differentiated human bone marrow cells and maximised bone formation within the scaffold as well as integration with the host tissue. Mason trichrome and OPN immunostaining confirmed the osteoblastic differentiation and mineralised tissue formation throughout the entire thickness of the DSM/SCPC/hMSCs onlay sheet placed on the top of decorticoid bone or critical size defect in the calvaria. Colour deconvolution of the OPN staining showed maturation of the new bone as indicated by comparable intensity values to that of the non-operated natural bone. Areas of woven bone with dark OPN staining were observed at the centre of the critical size defect together with bone spicules containing osteocytes and haversian systems. Applying the DSM/SCPC/hBMSC as an onlay sheet in decorticoid defect resulted in a two to three times increase in bone thickness compared with that of non-operated bone. Interestingly, bone thickening was also noticed on the host bone next to the grafted defect because of the stimulating effect of SCPC granule dissolution on host bone cell activity. The later observation was confirmed with our previous work [2]. Collectively, the findings of the analysis indicate that the DSM/SCPC/hBMSC tissue engineering scaffold can be used for bone augmentation and tissue regeneration. It is also possible to use the DSM/SCPC/hBMSC as a drug delivery system to treat diseases involving hard and soft tissues. In literature it has been demonstrated the use of Resorbable Bioactive Ceramic like SCPC and Calcium sulphate/Hyroxyapaptite was useful in releasing Bone morphogenic Protein by adsorption on the surface of ceramic which allows slow release of active molecule over time [3, 26].

The fact that DSM has extracellular matrix proteins that are site-specific and give protein “footprints” of previous resident cells has prompted its use for soft-tissue regeneration. In addition, the DSM provided a biomimetic scaffold that can easily be revascularised once it’s placed in situ [6, 7]. Total muscle transition was observed, with tissue becoming a loose connective tissue consisting of osteoblasts and pre-osteoblasts. This observation was seen also in previous published paper muscle graft/or flap were used as scaffold at bone defect [2, 3]. It is also rational to imagine that osteogenesis follows the path of intramembranous ossification, and is triggered by the presence and transformation of mesenchymal stem cells (MSCs) into osteoblasts within the revascularized connective tissue matrix. This fact was consistent with our finding in a previous study in mandibular bone [2].

To augment the process of bone regeneration, we supplemented the DSM with a resorbable bioactive SCPC ceramic, and hMSCs successfully demonstrated the ability of the DSM/SCPC/hBMSC scaffold to regenerate and augment bone in two different surgical defect models. Bone healing took place without any complications, swelling, inflammation, or severe foreign body reaction. One may argue the use of nude mice would preclude the inflammatory process which may happened. The use of muscle DMS as a bioreactor and the use of SCPC have been tested before in animal model with no reported complication [26, 27].

The regenerated bone in the regions grafted with DSM/SCPC/hBMSC was highly vascularises with extensive cellular activities. Vascularisation is necessary for the viability and functioning of the newly formed tissue, more importantly for critical defect regeneration [12, 13]. Blood vessels and haversian systems were noted near the SCPC particles surrounded by the de novo bone. In conjunction with the presence of numerous blood vessels, there was a high cellular activity indicating efficient delivery of oxygen and nutrients necessary for the metabolic activity and the progress of new bone formation [28, 29]. It has been suggested that since the DSM is rich in vessels, it can easily be revascularised by migrated endothelial cells [6, 7]. However, in the present study, superior vascularisation was noticed in the presence of the SCPC. Previous studies have correlated between the silica ions released from bioactive SCPC ceramic and vascularisation of newly formed bone [26, 30, 31]. Li and Chang reported that Si ions stimulated the human umbilical vein endothelial cells to highly express vascular endothelial growth factors (VEGF) and subsequently activated kinase domain insert receptor by themselves to initiate the angiogenesis pathway [32]. Therefore, the enhanced angiogenesis of the regenerated bone in the DSM/SCPC/hBMSC grafted regions is synergized by the silicon ions released from the SCPC resorbable bioactive ceramic.

It should be noted that many studies have used angiogenic growth factors like VEGF or basic fibroblast growth factor to enhance angiogenesis [33]. However, the delivery of growth factors by polymeric scaffolds carries many limitations related to the loss of molecular activity during incorporation in the carrier and the risk of carcinogenicity associated with uncontrolled delivery [34, 35]. Therefore, the ability for the DSM/SCPC/hBMSC to enhance angiogenesis of mineralised tissue without incorporating any growth factor is a major advantage over scaffolds incorporating growth factors.

With respect to bone regeneration, the regenerated bone within the muscle flap was associated to the undegraded residual SCPC, which has contributed significantly in initiating bone formation. Also it is doubtful that rMSCs may have induced bone regeneration in the DMS. The bone detected was at different stages of maturation; the more mature bone with a lamellar structure was observed at the periphery, and an area of woven bone was seen toward the centre. Moreover, less mature osteoid bone was detected at the centre of the defect, situated close to the periosteum-like loose connective tissue. The later observation was confirmed with dynamic histomorphometrics in our previous work [2].

In addition, the SCPC stimulated bone tissue formation by virtue of its bioactive property. Bone cells attached directly on the surface of SCPC particles and deposited bone. Mason trichrome and OPN immunostaining confirmed the mineralisation of the newly formed bone. Bone formation was observed interlinking with host bone, as islands within the DSM/SCPC/hBMSC graft. The SCPC is a balanced O/Si/Ca/Na/P ceramic recipe with multiple mechanisms for enhancement of new bone formation. The controlled dissolution of SCPC in physiological solutions and their effects on osteogenic gene expression have been studied extensively [35–37].

Silica gives the impression of being necessary for bone regeneration, as well as related with both the organic and inorganic phases of bone [38]. The release of SCPC ions such as Si, Na and Ca stimulates osteoblast function and downregulates osteoclast activity. Differentiated osteoblasts require plenty of Ca ions to produce mineralised tissue. Under normal conditions, osteoblasts get the Ca supply from the blood.

However, in large defects and inside a 3D tissue engineering scaffold, the shortage of blood supply would prohibit efficient mineralisation. Therefore, the SCPC provided an exogenous source of Ca and P to support mineralisation [25]. The mineralisation of the bone matrix was evident inside the DSM/SCPC/hBMSC scaffold and in the centre of the critical size defect.

Of prime importance is the increase in bone thickness and the number of bone marrow spaces at the host bone adjacent to the grafted defect. Bone is a dynamic tissue containing osteoblasts and osteoclasts responsible for its remodelling [39]. Mechanical loading of bone stimulates osteoblast activity and results in bone thickening [40]. Conversely, the absence of mechanical signal facilitates osteoblastic activity and bone resorption. The calvarial bone defect models used in our study is non-load-bearing. Therefore, the observed bone thickening cannot be explained by trans mechanical effect. Rather, it is explained by the signalling effect of the dissolved ceramic ions that diffuse into the host bone and modulate remodelling and the role of biomimetic scaffolding with the presence of osteogensis. The diffusion of ceramic ions was motivated by the concentration gradient in the tissue fluids. Studies have showed that dissolved SCPC ions promote osteoblastic differentiation of bone marrow cells [17, 23, 24]. In addition, SCPC dissolution products have demonstrated to decrease the bone resorption capacity of osteoclasts. High-performance gene sequence and pathway research associated with SCPC dissolution products and improved expression of essential osteogenic factors and phenotypic markers liable for bone cell signalling, differentiation, mineralised ECM synthesis, cell-cell and cell-ECM reactions [41]. Signalling MSC by dissolved ions from SCPC appeared to be more practical and safer than other approaches that used bone morphogenetic protein 2 (BMP-2) or combined progenitor cells accompanied by gene therapy to regenerate bone. Literature evidence indicated that the recombinant human BMP-2 in addition to its capacity to facilitate the expression of osteogenic genes, also induces bone resorption markers and inflammatory cytokines [42–44]. To overcome these risk factors, a systemic administration of anti-resorptive agents like nuclear factor-kB ligand receptor activator (RANKL) antagonists, bisphosphonates, or antisclerostin antibodies was investigated [45–47]. Deng et al. have shown that the collapse of miR-31 facilitates osteogenesis of BMSCs [48]. Genetic engineering of the cells involved the construction and transduction of lentiviral vectors encoding negative power, miR-31 precursors, and anti-sense sequences.

The treatment of critical size calvarial defects with a scaffold seeded with miR-31-modified BMSCs showed new bone formation with an ~60% regeneration rate. Our approach to induce bone regeneration relied on signalling the infused hMSCs by the dissolved ions of SCPC resorbable bioactive ceramic. Our previous in vitro work [7] provided evidences that the SCPC incorporated in DSM provided osteogenesis cues to hMSCs leading to upregulation of gene expression of bone markers including OCN and OPN. hMSCs seeded on DSM in the absence of SCPC upregulated bone sialoprotein associated with cell proliferation [7]. The upregulation of OCN and OPN expression by SCPC confirms its important role in promoting osteogenesis within the DSM/SCPC scaffold and at the interface with host bone. Along with the increased thickness of bone, we observed an increase in the number of marrow spaces in clavaria grafted with DSM/SCPC.

The MSCs are essential for the homoeostasis of bone. Previous assessments have examined the influence of calcium ions (Ca2+) release from bone in the time of osteoclast-mediated bone resorption on MSC activity nearby bone resorption sites. [49]. Elevated extracellular Ca2 + has been found to facilitate cell proliferation and migration, as well as upregulation of OPN production and matrix mineralisation. Similar effects of Ca^2+^ concentration on MSCs phenotypic expression and differentiation have been reported [50–54]. In our study, we observed increased OPN staining at the interface between the DSM/SCPC/hBMSC graft and the host bone. Therefore, the increased number of bone marrow cells in bone adjacent to the DSM/SCPC could be attributed to the local increase in Ca^2+^ ions dissolved from the SCPC and diffused to the host bone. The ability of the DSM/SCPC to recruit exogenous and stimulate introgenous hBMSC proliferation, differentiation and new bone formation points to its potential application in bone augmentation and treatment of several skeletal pathologies including osteoporosis.

The limited number of operated cases, and thus limited study groups, were the shortcomings of our study. Preferably, the impact of hBMSC-seeded DSM and SCPC/DSM needed to be investigated in bigger samples. Yet, we abide by animal ethics and 3R (reduce, refine, and replace) by laws for animal safety. Our pervious, controlled trial showed that the experimental group that was filled with muscle grafts and bioglass material reported active bone remodelling, but does not fill the defect [5].

The present study demonstrates the mechanism of bone augmentation and bone regeneration at the cellular level in two different study designs. In addition, related experiments may be conducted in large animal samples utilising a virtual model such as a stress-bearing area like long bone or jaw bone. Probably carrying out similar experiment in long bone would give more information due to its superior quantity of cortical and cancellous bone and different path of ossification. However, the repair of cranial defects is a challenge for cranio-facial surgeons, and detailed trials are ongoing for tissue engineering of calvarian bone defects. [55]. In addition, this anatomical location was chosen, as we primarily wanted to highlight the significance of using decellularised muscle graft for bone regeneration. At the same time this area being easily accessible (cranial defect) for small animal models, helped us to improve our operating skills with limited animal distress on the basis of recommendations for the use of animals in clinical study.

## Conclusion

The DSM/SCPC/hBMSC construct promoted bone regeneration in decorticoid and critical size calvarial defects. The regenerated bone in the defect or in the remnants of the DSM matrix was highly vascularised and contained osteocytes and haversian systems. Mason trichrome and OPN staining confirmed osteoblast differentiation and tissue mineralisation. Engineering the DSM matrix with SCPC resorbable bioactive bone graft induced osteoblast activity inside the defect as well as in the host bone, leading to a substantial improvement in bone thickness. The results of this study suggest potential use of the DSM/SCPC tissue-engineered platform for bone regeneration.
